# Structure-Activity Relationship Studies on the Macrolide Exotoxin Mycolactone of *Mycobacterium ulcerans*

**DOI:** 10.1371/journal.pntd.0002143

**Published:** 2013-03-28

**Authors:** Nicole Scherr, Philipp Gersbach, Jean-Pierre Dangy, Claudio Bomio, Jun Li, Karl-Heinz Altmann, Gerd Pluschke

**Affiliations:** 1 Molecular Immunology, Swiss Tropical and Public Health Institute, Basel, Switzerland; 2 University of Basel, Basel, Switzerland; 3 Department of Chemistry and Applied Biosciences, Institute of Pharmaceutical Sciences, Swiss Federal Institute of Technology (ETH) Zürich, Zürich, Switzerland; University of Tennessee, United States of America

## Abstract

**Background:**

Mycolactones are a family of polyketide-derived macrolide exotoxins produced by *Mycobacterium ulcerans*, the causative agent of the chronic necrotizing skin disease Buruli ulcer. The toxin is synthesized by polyketide synthases encoded by the virulence plasmid pMUM. The apoptotic, necrotic and immunosuppressive properties of mycolactones play a central role in the pathogenesis of *M. ulcerans*.

**Methodology/Principal Findings:**

We have synthesized and tested a series of mycolactone derivatives to conduct structure-activity relationship studies. Flow cytometry, fluorescence microscopy and Alamar Blue-based metabolic assays were used to assess activities of mycolactones on the murine L929 fibroblast cell line. Modifications of the C-linked upper side chain (comprising C12–C20) caused less pronounced changes in cytotoxicity than modifications in the lower C5-O-linked polyunsaturated acyl side chain. A derivative with a truncated lower side chain was unique in having strong inhibitory effects on fibroblast metabolism and cell proliferation at non-cytotoxic concentrations. We also tested whether mycolactones have antimicrobial activity and found no activity against representatives of Gram-positive (*Streptococcus pneumoniae*) or Gram-negative bacteria (*Neisseria meningitis* and *Escherichia coli*), the fungus *Saccharomyces cerevisae* or the amoeba *Dictyostelium discoideum*.

**Conclusion:**

Highly defined synthetic compounds allowed to unambiguously compare biological activities of mycolactones expressed by different *M. ulcerans* lineages and may help identifying target structures and triggering pathways.

## Author Summary

Buruli ulcer is a chronic necrotizing skin disease caused by *Mycobacterium ulcerans*. The characteristic histopathological features of Buruli ulcer, severe destruction of subcutaneous tissue with minimal inflammation in the core of the lesion, are primarily attributed to the cytotoxic activity of mycolactone, the macrolide exotoxin of *M. ulcerans*. Different geographical lineages of *M. ulcerans* produce different structural variants of mycolactone. By using highly defined synthetic mycolactones, including both naturally occurring molecular species and additional non-natural variants, we have assessed the influence of the structure of the C-linked upper side chain and the lower C5-O-linked polyunsaturated acyl side chain on biological activity. Changes in the lower side chain affected the cytotoxic activity against mammalian cells more profoundly than changes in the upper side chain. Mycolactone A/B had no antimicrobial activity against Gram-positive and Gram-negative bacteria and was also inactive against *Saccharomyces* and *Dictyostelium*.

## Introduction

The macrolide exotoxin mycolactone is a key virulence factor of *M. ulcerans* and plays a central role in the pathogenesis of Buruli ulcer [1]. Mycolactones have been shown to act both *in vivo* and *in vitro* on various mammalian cell types, including fibroblasts [1]–[6], adipocytes [7], keratinocytes [8], myocytes [9], [10], macrophages [2], [6], [11]–[14], dendritic cells [15] and T-cells [16]–[18]. Effects caused by mycolactones include induction of apoptosis/necrosis, cytoskeletal rearrangements, impaired cytokine production and interference with cellular signaling.

The polyketide synthases required for mycolactone biosynthesis are encoded on the extrachromosomal plasmid pMUM [19], [20]. *M. ulcerans* has evolved from a common *M. marinum* ancestor by acquisition of this plasmid and has subsequently diverged into two principal mycolactone-producing lineages [21], [22]. The “classical” lineage includes *M. ulcerans* isolates associated with Buruli ulcer from Africa and Australia. The “ancestral” lineage includes both Buruli ulcer isolates from Japan, China and Mexico and isolates from fish and frogs previously also designated *M. pseudoshottsii*, *M. liflandii* or *M. marinum* [23], [24]. Mycolactones are composed of a 12-membered macrolide core and two attached side chains; a short upper, C-linked side chain (comprising C12–C20) and a longer lower, C5-O-linked polyunsaturated acyl side chain. While the macrolide core structure and upper side chain are conserved, mycolactone populations from different *M. ulcerans* sub-lineages vary in the length, the number and localization of hydroxyl groups and in the number of double bonds of the lower side chain. *M. ulcerans* strains may produce several molecular variants of mycolactone, with one or more species dominating [25]. The mycolactone repertoire seems to be highly conserved within a defined geographical sub-lineage of *M. ulcerans* [25]. Mycolactone A/B is produced by strains of the classical *M. ulcerans* lineage found in Africa and is regarded as the most potent toxin. Australian classical lineage strains produce - in addition to mycolactone A/B - mycolactone C, which lacks one hydroxyl group. Mycolactone D with an additional methyl group is produced by Chinese strains belonging to the ancestral lineage. *M. ulcerans* ancestral lineage isolates from fish and frogs have been found to produce the mycolactone variants E and F.

Mycolactones have previously been prepared from *M. ulcerans* cultures by a two-step extraction procedure, yielding preparations of acetone soluble lipids predominantly containing mycolactone. These extracts can be further purified by chromatographic methods [26]; nevertheless, the use of extracted mycolactones for comparative studies may be compromised by the heterogeneity of preparations. Therefore, biological studies with highly defined synthetic mycolactones represent an attractive alternative. Based on the established synthesis of the mycolactone core [27]–[30], different synthesis strategies have been pursued for the stereoselective partial and total synthesis of mycolactones [31]–[33]. In addition, simplified C8-desmethyl-mycolactone analogues have been synthesized, which were analyzed for their cytopathic potency by using cell rounding as a parameter to compare cytotoxic activities [34]. No systematic structure-activity relationship studies on larger sets of synthetic mycolactones have been published so far. Results of individual studies cannot be reliably compared, since different readouts, such as cell rounding [22], [34], [35], cytokine production [36] or flow cytometric parameters [35], [36] have been employed. Furthermore, different cell lines (such as Jurkat T-cells [36], murine fibroblasts [22], [34], [35] and sets of human tumor cell lines [37] have been used and cytotoxic activity has been assessed after different times, such as 24 hours [36] or 24 and 48 hours [35]. Most of these structure-activity studies have been limited to mycolactone A/B and a limited number of derivatives. A comparison of the activity of eight C8-desmethyl mycolactone analogues is hampered by the fact that lack of the C8-methyl substituent reduces the cytopathic activity by a factor of 125 [34]. Here, we have performed more systematic comparative studies using synthetically produced natural toxins and additional structural mycolactone variants that are not found in nature.

## Materials and Methods

### Synthetic mycolactones

We recently reported the synthesis of mycolactone A/B [38]. Details of the syntheses of mycolactones C and F and of the six non-natural mycolactone derivatives will be published elsewhere (Gersbach *et al.*, manuscript in preparation). Briefly, all mycolactones discussed here were prepared by the same overall strategy that we had previously developed for the synthesis of mycolactone A/B. Thus, a modified Suzuki coupling was employed to establish the C12–C13 bond and elaborate the full upper side chain and a Yamaguchi type acylation reaction was used to attach the lower side chain. All final products used for biological testing were purified by RP-HPLC; they were generally obtained as mixtures of (interconverting) double bond isomers. Analytical data for the synthetic compounds are provided as supplementary information (S1).

For biological testing, 0.5 mg/ml stock solutions of the mycolactones were prepared in cell culture grade DMSO (Sigma). Stock solutions were aliquoted and stored frozen at −20°C.

### Cytotoxicity assay

**Figure 1:**
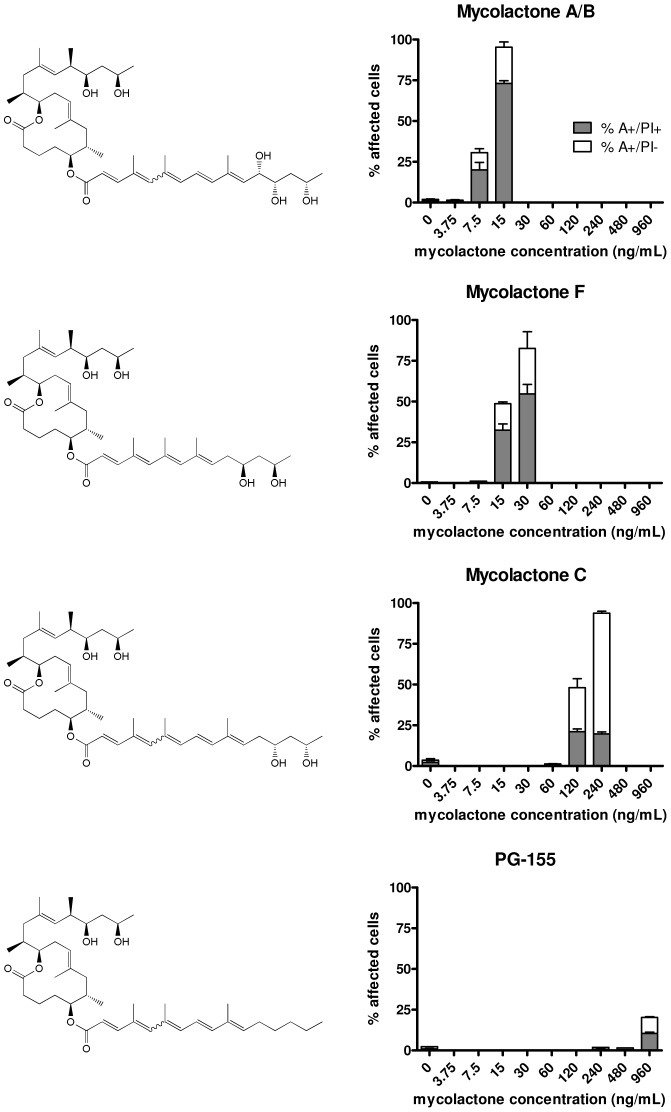
Cytotoxicity of mycolactone variants with modifications in the lower side chain. Cells were treated for 48 hours with different concentrations of mycolactone A/B, mycolactone F, mycolactone C or PG-155 and stained with annexin-V-FITC and PI. Flow cytometry was used to determine annexin-positive (A^+^) and PI-positive (PI^+^) cell populations. Triplicate samples were analyzed and mean values as well as standard deviations are shown.

**Figure 2:**
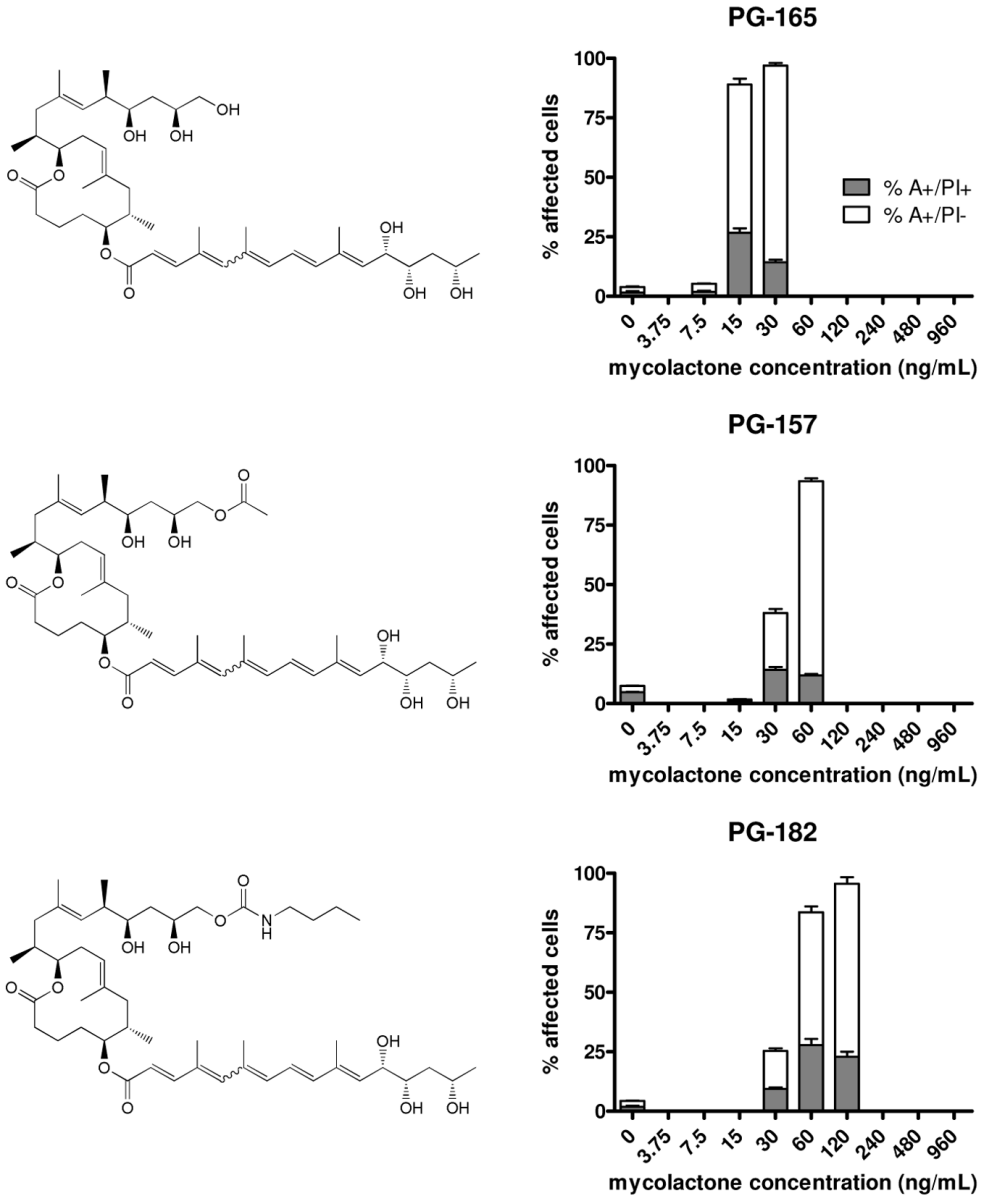
Cytotoxicity of mycolactone variants with modifications in the upper side chain. Cells were treated for 48 hours with different concentrations of PG-157, PG-165 or PG-182 and stained with annexin-V-FITC and PI. Flow cytometry was used to determine the annexin-positive (A^+^) and PI-positive (PI^+^) cell populations. Triplicate samples were analyzed and mean values as well as standard deviations are shown.

**Figure 3:**
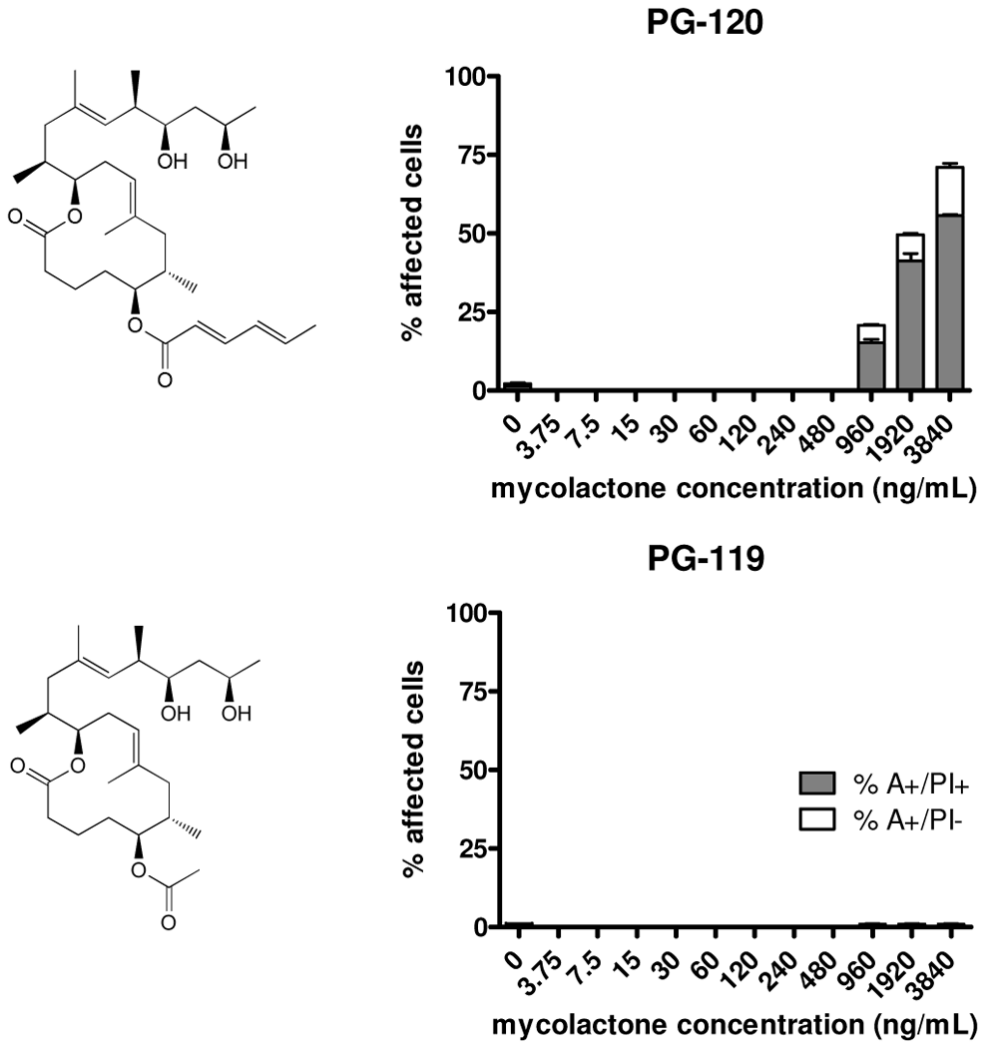
Cytotoxicity of truncated mycolactone variants. Cells were treated for 48 hours with different concentrations of PG-119 or PG-120 and stained with annexin-V-FITC and PI. Flow cytometry was used to determine the annexin-positive (A^+^) and PI-positive (PI^+^) cell populations. Triplicate samples were analyzed and mean values as well as standard deviations are shown.

Murine L929 fibroblasts were grown in RPMI medium (Gibco) supplemented with 10% FCS (Sigma), 2 mM glutamine (Gibco) and 0.05 mM β-mercaptoethanol (Gibco) and incubated at 37°C and 5% CO_2_. Cells were passaged 3–6 times prior to use in cytotoxicity experiments. For flow cytometry analysis 24,000 cells were seeded into 24-well plates (Falcon) and allowed to adhere o/n. Medium was then aspirated and replaced by 500 µl medium containing different concentrations of mycolactone and 0.12% DMSO (vol/vol). After incubation for 24, 48 or 72 h, cells were detached from the culture plates by repeated gentle flushing through a pipette tip without use of Trypsin-EDTA. Harvested cells were centrifuged for 10 min at 1,200*x*g, resuspended in 300 µl binding buffer with 0.2 µg/ml Annexin V-FITC (AnnexinV kit, Calbiochem) and incubated for 30 min at 4°C. The cells were spun again and pellets were resuspended in 300 µl staining buffer containing 0.3 µg/ml propidium iodide (AnnexinV kit, Calbiochem). Cell suspensions were analyzed by flow cytometry using a BD FACS Calibur Flow Cytometer (Becton Dickinson) and apoptotic (A^+^/PI^−^) and necrotic (A^+^/PI^+^) cell populations were determined using the CellQuest Pro Software (Becton Dickinson). The experiments were set up in triplicates and performed at least twice. Mycolactone A/B in a concentration range of 3.75 to 120 ng/ml was included as control in all experiments. The mycolactone concentration at which 50% of the cells were killed (LC_50_) was determined by plotting the percentage of affected cells (sum of A^+^/PI^−^ and A^+^/PI^+^ cells) against the log concentration of the individual mycolactones. In Fig. 1–3 only data for concentrations close to the LC_50_ are shown.

### Inhibition of cell proliferation

To measure proliferation of L929 fibroblasts, 24,000 cells were seeded into 24-well plates (Falcon) and allowed to adhere o/n. The medium was aspirated and replaced by 500 µl medium containing 60 ng/ml of mycolactone and 0.06% DMSO (vol/vol). At time point 0 and after 24, 48 and 72 h fibroblasts were harvested, diluted 1∶100 in isotonous solution and measured using an automated cell counting device (Casy®TT, Schärfe System). The experiment was set up in triplicates and performed twice.

### Microscopic analysis

24,000 cells were seeded on four-chamber glass slides (BD Falcon) with complete RPMI medium and allowed to adhere for 24 hours. The medium was aspirated and replaced by 500 µl medium containing different concentrations of mycolactone. After the specified incubation period, cells were washed once in PBS and fixed with 4% formaldehyde (Medite) for 20 min. Fibroblasts were washed again in PBS prior to permeabilization in Triton X-100 (0.1% in PBS) for 20 min. Cells were rinsed in PBS and blocked by incubation in 4% FBS in PBS for additional 20 min. The actin cytoskeleton was stained by incubating the cells for 1 h at room temperature with Texas Red-X phalloidin (3 units/ml, in blocking solution, Molecular Probes). Cells were washed in blocking buffer, then in PBS. ProLong Gold antifade reagent (Invitrogen) containing diamidino-2-phenylindole (DAPI) was used for nuclear counterstaining. Cover slips were mounted onto the slides and cell rounding as well as the staining of nuclei and actin cytoskeleton was qualitatively analyzed by fluorescence microscopy (Leica DM 5000 B). Mycolactone-induced changes in the pattern and intensity of the Texas Red-X phalloidin staining of the cytosolic actin cytoskeleton as well as in the uniform, round and clear-edged DAPI staining of nuclei in healthy cells were observed.

### Alamar Blue – based analysis of metabolic activity

Metabolic activity of mycolactone-treated L929 fibroblasts was analyzed by performing Alamar Blue assays. Seeding of cells and addition of mycolactones were performed as described for the flow cytometry-based cytotoxicity assays. After mycolactone treatment, alamarBlue® reagent (Invitrogen) was added to the wells (10% v/v) and the cells were further incubated for 1 hour at 37°C and 5% CO_2_. Fluorescence intensities were measured using a SpectraMax Gemini XS (Molecular Devices) and the values were calculated referring to the DMSO control (0 ng/ml mycolactone) set at 100%. The experiments were set up in triplicates and performed at least twice. The concentration at which the metabolic activity of cells was inhibited by half (IC_50_) was determined by plotting the fluorescence intensity against the log concentration of the individual mycolactones.

### Assessment of the antimicrobial activity of mycolactone A/B

Antimicrobial activity of mycolactone on *Streptococcus pneumoniae* (SP1, P1577), *E. coli* (DE(3)) and *Saccharomyces cerevisiae* was tested by applying the disk agar diffusion (Kirby-Bauer) method. Bacteria were pelleted, resuspended in PBS and spread on blood agar/LB agar. The plates were dried for 30 min and sterile paper disks were distributed circle-like onto the agar. Mycolactone A/B solutions of different concentrations (0.003 µg/ml to 10 µg/ml) were applied on the paper disks (40 µl). The agar plates were incubated o/n at 37°C and then analyzed for potential zones of inhibition.

The effect of mycolactone A/B on the growth of *Dictyostelium discoideum* DH1-10 was assessed by performing an Alamar Blue assay in a 24-well format. 2,000 cells were seeded in 500 µl medium containing mycolactone in the concentration range of 0.16 to 500 ng/ml. As controls, DMSO and blasticidin were used. After an incubation period of 3 days at room temperature, alamarBlue® reagent (Invitrogen) was added and the plate was further incubated for 18 hours at room temperature.

## Results

Recently we described a novel strategy for the synthesis of mycolactone A/B that is based on the stereoselective construction of the macrolactone core by ring-closing olefin metathesis and subsequent incorporation of the C- and O-linked side chains by suitable fragment couplings [38]. Taking this synthesis approach a set of natural mycolactones (mycolactone A/B, mycolactone C, mycolactone F) and additional derivatives displaying modifications in the lower or upper side chain (PG-119, PG-120, PG-155, PG-157, PG-165 and PG-182) were produced (see Figures 1, 2 and 3) for biological testing. The biological activity of these synthetic compounds on the murine L929 fibroblast cell line was assessed by flow cytometry. After treatment with different concentrations of synthetic mycolactones, cells were stained both with FITC-labeled annexin-V and with propidium iodide. Annexin-V binds to exposed phosphatidylserine residues translocated from the inner to the outer leaflet of the plasma membrane in cells undergoing apoptosis. Propidium iodide intercalates into the DNA of cells that have lost nuclear membrane integrity, serving as a marker for necrosis. Quadrant analysis was performed to determine apoptotic (A^+^/PI^−^) and necrotic (A^+^/PI^+^) cell populations.

While first signs of mycolactone A/B-mediated cell death were already detectable after 24 hours, significant effects were only observed after 48 hours [38]. For comparison with mycolactone A/B, the lethal concentration of mycolactone analogues at which 50% of the cells were affected (LC_50_) was therefore determined after 48 hours (Table 1). As described previously [38], mycolactone A/B was highly potent (Figure 1) with a LC_50_ of 12 nM (Table 1). For the two naturally occurring structural variants mycolactone F and mycolactone C, the LC_50_ values were 29 nM and 186 nM, respectively (Table 1). Mycolactone C differs from mycolactone A/B in lacking the hydroxyl group at position C12 of the lower side chain. Mycolactone F has a shorter side chain with also only two hydroxyl substituents (Figure 1). While these natural mycolactones retained cytotoxic activity, compound PG-155, a non-natural structural variant devoid of all hydroxyl groups in the lower side chain, showed only minor activity with a LC_50_ of 4550 nM (Table 1).

**Table 1:** Comparison of LC_50_ and IC_50_ values of the mycolactone variants.

Mycolactone	MW (g/mol)	LC_50_ (nM)	IC_50_ (nM)	LC_50_/IC_50_
Mycolactone A/B	743	12	5	2.4
Mycolactone F	701	29	9	3.2
Mycolactone C	727	186	122	1.5
PG-155	695	4550	1439	3.2
PG-165	759	15	5	3.0
PG-157	801	45	20	2.3
PG-182	858	50	16	3.1
PG-119	467	≫5000	≫5000	n.a.
PG-120	519	3426	171	20

Apart from these mycolactone variants with modifications in the lower side chain, also analogues with modifications in the upper side chain were synthesized and tested (Figure 2). Introduction of a hydroxyl group at C20 in compound PG-165 had no major effect, since PG-165 had only a slightly higher LC_50_ (15 nM) than mycolactone A/B (Figure 2, Table 1). In addition, derivatisation of this hydroxyl group into an acetate (PG-157 with a LC_50_ of 45 nM) or into a bulky butyl carbamate (PG-182 with a LC_50_ of 50 nM) reduced cytotoxicity only about three-fold (Figure 2, Table 1). Thus, the upper side chain turned out to be relatively tolerant to a significant extension in length and to the presence of polar linker elements between the natural side chain and the extension module.

**Figure 4:**
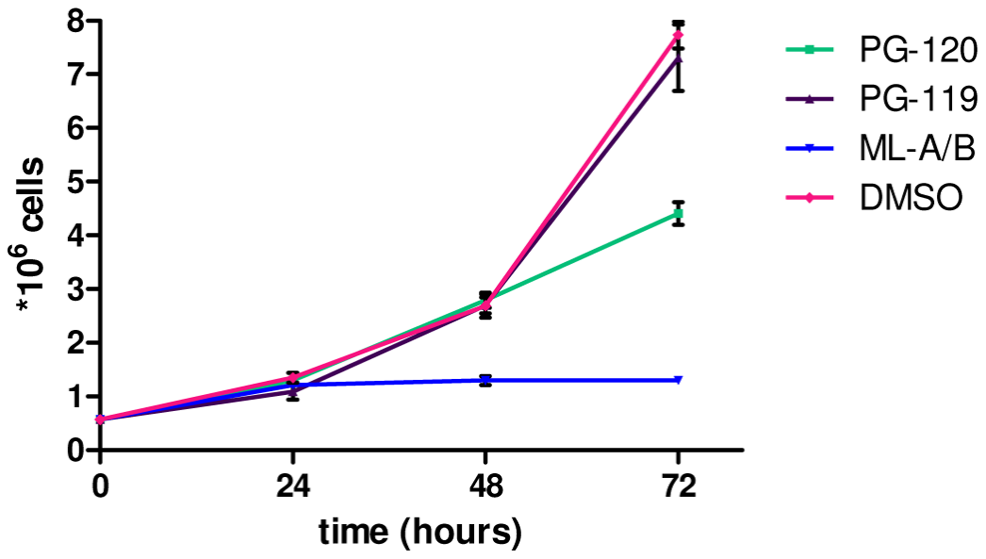
Inhibition of proliferation of L929 fibroblasts upon treatment with PG-120, PG-119 and mycolactone A/B. Cell proliferation was measured after 0, 24, 48 and 72 h by determining the number of cells treated with 60 ng/ml of PG-120, PG-119 or mycolactone A/B. Mean values and standard deviations of triplicates are shown.

**Figure 5:**
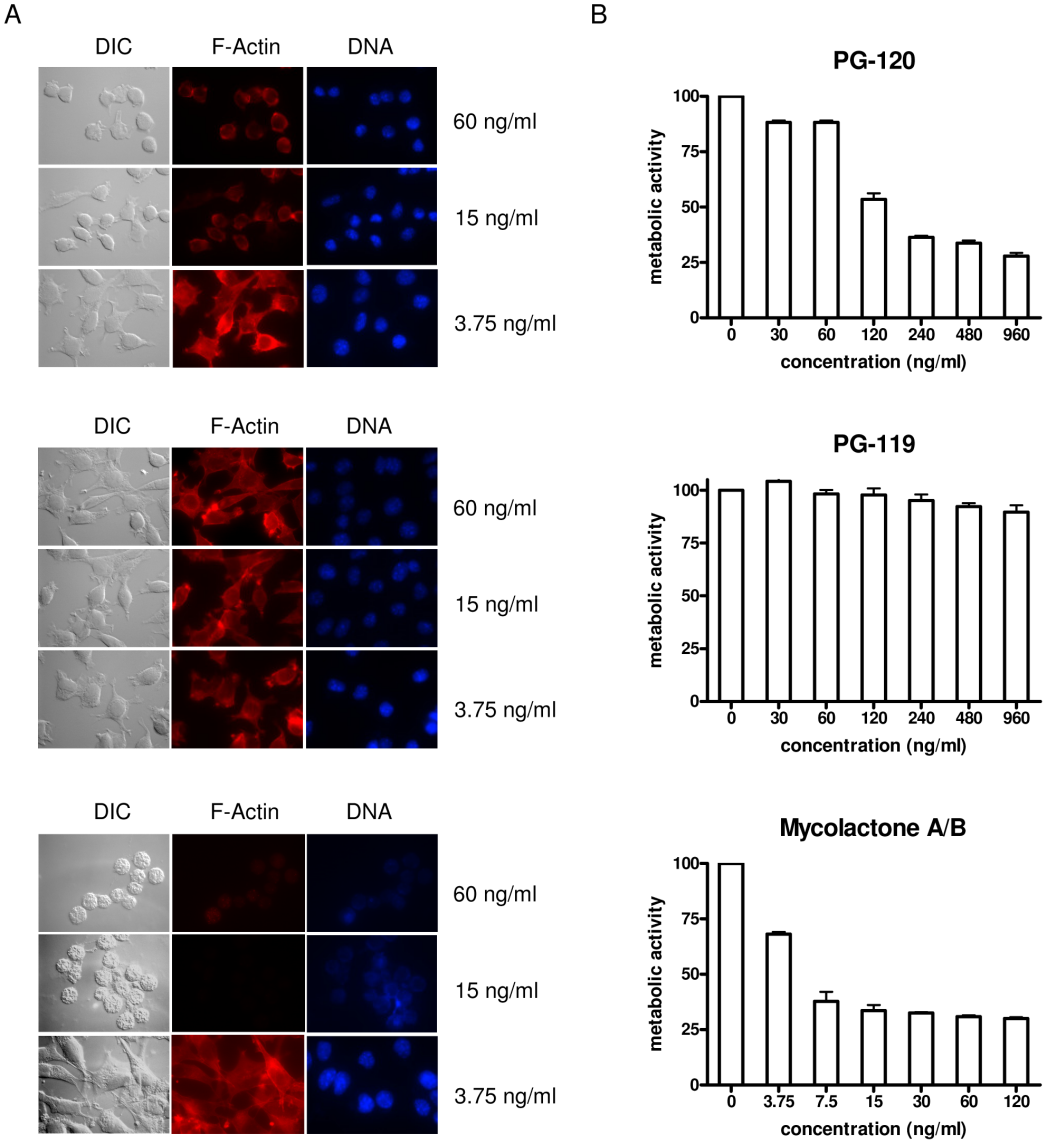
Changes in morphology and metabolic activity of L929 fibroblasts upon treatment with PG-120, PG-119 and mycolactone A/B. (A) For microscopic analysis, cells incubated for 48 h with selected concentrations of mycolactones were stained with phalloidin to label the actin cytoskeleton (red) and with DAPI (blue) to visualize the nuclei. The slides were analyzed by fluorescence microscopy using a 787-fold magnification. (B) For the analysis of metabolic activity, alamarBlue® reagent was added to fibroblasts incubated for 48 hours with different concentrations of PG-120, PG-119 or mycolactone A/B. Fluorescence intensities of triplicate samples were measured and mean values as well as standard deviations are shown.

PG-120, a derivative with a significantly truncated lower side chain, showed some residual cytotoxic activity (LC_50_ = 3426 nM), whereas PG-119, a derivative with an acetyl residue as the lower side chain, showed no activity within the concentration range tested (Figure 3, Table 1). For all compounds, except PG-120, concentrations required for cytotoxic activity (as measured by flow cytometry), reduction of metabolic activity in an Alamar Blue-based assay, changes in the intensity and pattern of phalloidin-staining of the actin cytoskeleton and changes in the round, clear-edged and uniformly stained nuclear morphology of normal cells were in the same range. While the IC_50_ value for PG-120 (171 nM; Table 1), was twenty-fold lower than the LC_50_, the LC_50_/IC_50_ ratios of all other compounds with widely varying toxic potency ranged between 1.5 and 3.2 (Table 1). Furthermore, at such sub-lethal PG-120 concentrations a marked reduction in cell proliferation (Figure 4), and a transient effect on the actin cytoskeleton accompanied by the rounding up of the cells, without changes in nuclear morphology was observed (Figure 5A). A similar activity was not observed for PG-119 (Figures 4 and 5).

When analyzed for antimicrobial activity, mycolactone A/B was found inactive against all microbial species tested, including Gram-positive (*Streptococcus pneumoniae*) and Gram-negative (*Neisseria meningitis*, *Escherichia coli*) bacteria; it was also inactive against yeast (*Saccharomyces cerevisae*) and amoeba (*Dictyostelium discoideum)*.

## Discussion

Our flow cytometric analyses of murine fibroblast L929 cells treated with a series of synthetic mycolactones reconfirmed that changes in the O-linked lower side chain can profoundly affect the biological activity. Activity of the synthetic mycolactone A/B was in the range reported for mycolactone preparations extracted from *M. ulcerans* cultures [1]. Mycolactone F was about two times less active and mycolactone C about 15 times less active than mycolactone A/B, respectively. For extracted mycolactone C an even far more pronounced difference in activity compared to mycolactone A/B has been described in assays determining L929 fibroblast rounding at 24 h and loss of monolayer at 48 h [25]. In addition to mycolactone C, Australian *M. ulcerans* strains also produce mycolactone A/B. Our data indicate that this mycolactone A/B portion may be more important for the pathogenesis caused by these strains than mycolactone C. In accordance with our findings, only a slightly lower activity was observed, when extracted mycolactone F was compared to mycolactone A/B in a L929 cell apoptosis assay at 24 h [3]. When the inhibition of IL2 production by activated Jurkat T-cells instead of cell death was used as readout, both mycolactones F and C were dramatically less potent than mycolactone A/B [36].

While we have investigated different types of modifications for the lower and upper side chains, it is clear that both the incorporation of polar substituents at C20 and the extension of the upper side chain by up to 7 heavy atoms, in contrast to most of the modifications of the lower side chain, does not lead to a substantial loss in cytotoxicity. It remains to be seen how the removal of hydroxyl groups from the upper side chain or its overall shortening would affect potency.

It has been proposed that mycolactones enter mammalian cells via passive diffusion and interact with cytosolic target(s) [39]. Reduced or abolished activity of structural variants of mycolactone may thus be related to lack of binding to target structure(s), inefficient triggering of activation pathways or reduced translocation across the cell membrane. Studies using isotopically labeled rather than fluorescence labeled structures with altered biophysical properties are required to gain better insight into mechanisms that allow mycolactones to cross biological membranes.

Our findings with the truncated mycolactone PG-120 shows that different biological effects of mycolactone can be dissociated by using structural variants. In line with these observations, sub-lethal doses of mycolactone A/B have been shown to alter trafficking and cytokine production of lymphocytes and macrophages [40], [41]. It remains to be elucidated whether different pathways and target structures are involved in the triggering of the biological effects of mycolactone.

Since a number of macrolides have antibiotic activity against a broad spectrum of bacteria it has been speculated that mycolactone secreted by *M. ulcerans* during active disease may prevent superinfection of BU wounds. However, synthetic mycolactone A/B showed no antimicrobial activity against any of the microorganisms tested here. In line with this observation, superinfection of Buruli ulcer lesions seems to be much more common than traditionally anticipated (Yeboah-Manu et al., personal communication).

Much has still to be learnt about the biophysical properties of mycolactones, their distribution and stability in biological systems, their target structures and triggering pathways in mammalian cells. Synthetic natural mycolactones, isotopically labeled derivatives and structural variants represent valuable tools to address these open questions in future.

## Supporting Information

Dataset S1 Analytical data of the synthetic mycolactone variants used in the study. Nuclear magnetic resonance and mass spectrometry analysis were performed in order to confirm identity and purity of the individual mycolactones.
(DOCX)
