# Supplementary material for: Structure-Activity Relationship Studies on the Macrolide Exotoxin Mycolactone of Mycobacterium ulcerans
Source: PLoS Negl Trop Dis. 2013 Mar 28;7(3):e2143. doi: 10.1371/journal.pntd.0002143 (PMC3610637; doi:10.1371/journal.pntd.0002143)
Supplement: Dataset S1 — Analytical data of the synthetic mycolactone variants used in the study. Nuclear magnetic resonance and mass spectrometry analysis were performed in order to confirm identity and purity of the individual mycolactones. (DOCX) [file pntd.0002143.s001.docx]

# Dataset S1

NMR spectra were recorded on a Bruker AV-400 400 MHz and a Bruker DRX-500 500 MHz spectrometer at room temperature. Chemical shifts are reported as *δ* values (ppm). The solvent peaks were used as internal standards: Chloroform (*δ* 7.26), or acetone (*δ* 2.05) for ^1^H spectra, and chloroform (*δ* 77.0), or acetone (*δ* 29.84) for ^13^C spectra. Data are reported as follows: s = singlet, d = doublet, t = triplet, q = quartet, m = multiplet, br = broad signal. For inseparable isomers, ^1^H-NMR and ^13^C-NMR signals were assigned by HSQC and HMBC experiments. Infrared spectra (IR) were recorded on a Jasco FT/IR-6200 spectrometer. The absorption bands are given in wave numbers (cm^-1^). Optical rotations were measured on a Jasco P-1020 polarimeter. Mass spectra were recorded on a Waters Micromass AutoSpec Ultima (EI-Sector) or a Varian IonSpec Ultima (MALDI/ESI-FT-ICR) (both MS service of Laboratory of Organic Chemistry (LOC) at the ETH Zurich).

# Characterizations of Mycolactone Derivatives

**Mycolactone C.** *R_f_* (EtOAc) = 0.50; ***Z*-Δ^4’,5’^ isomer:** ^1^H-NMR (500 MHz, acetone-d6): *δ* 7.93 (d, *J* = 15.7 Hz, 1H), 6.67 – 6.27 (m, 3H), 6.14 (d, *J* = 10.8 Hz, 1H), 5.92 (d, *J* = 15.7 Hz, 1H), 5.77 – 5.67 (m, 1H), 5.20 – 5.07 (m, 1H), 5.03 (d, *J* = 9.7 Hz, 1H), 4.94 – 4.82 (m, 1H), 4.77 – 4.64 (m, 1H), 4.50 – 4.10 (m, 4H, OH), 4.04 – 3.80 (m, 3H), 3.55 – 3.44 (m, 1H), 2.58 – 2.27 (m, 5H), 2.19 – 1.34 (m, 16H), 2.01 (s, 3H), 1.97 (s, 3), 1.84 (d, *J* = 2.8 Hz, 3H), 1.71 (d, *J* = 8.2 Hz, 3H), 1.65 (s, 3H), 1.16 – 1.11 (m, 6H), 0.97 (d, *J* = 6.7 Hz, 3H), 0.94 – 0.79 (m, 6H); ^13^C-NMR (125 MHz, acetone-d6): *δ*173.3, 166.9, 143.2, 142.0, 140.6, 137.3, 136.5, 135.3, 134.4, 133.4, 131.9, 131.8, 131.2, 123.8, 123.4, 119.4, 79.3, 76.9, 76.3, 72.1, 68.9, 68.2, 46.4, 45.7, 44.3, 43.8, 40.5, 38.0, 36.0, 35.4, 32.7, 31.4, 29.7 (obscured by solvent signal), 24.6, 24.4, 21.0, 20.7, 20.6, 17.6, 17.2, 16.2, 15.9, 15.0, 12.8; ***E*-Δ^4’,5’^ isomer:** ^1^H-NMR (500 MHz, acetone-d6): *δ* 7.36 (d, *J* = 15.5 Hz, 1H), 6.67 – 6.27 (m, 4H), 5.88 (d, *J* = 15.5 Hz, 1H), 5.77 – 5.67 (m, 1H), 5.20 – 5.07 (m, 1H), 5.03 (d, *J* = 9.7 Hz, 1H), 4.94 – 4.82 (m, 1H), 4.77 – 4.64 (m, 1H), 4.50 – 4.10 (m, 4H, OH), 4.04 – 3.80 (m, 3H), 3.55 – 3.44 (m, 1H), 2.58 – 2.27 (m, 5H), 2.19 – 1.34 (m, 16H), 2.08 (s, 3H), 2.06 (s, 3H), 1.84 (d, *J* = 2.8 Hz, 3H), 1.71 (d, *J* = 8.2 Hz, 3H), 1.65 (s, 3H), 1.16 – 1.11 (m, 6H), 0.97 (d, *J* = 6.7 Hz, 3H), 0.94 – 0.79 (m, 6H); ^13^C-NMR (125 MHz, acetone-d6): *δ* 173.3, 166.9, 151.3, 144.6, 141.0, 137.3, 136.7, 136.5, 133.9, 133.4, 132.7, 132.2, 131.2, 123.8, 123.4, 117.1, 79.3, 76.9, 76.3, 72.1, 68.9, 68.2, 46.4, 45.7, 44.3, 43.8, 40.5, 38.0, 36.0, 35.4, 32.7, 31.4, 29.7 (obscured by solvent signal), 24.6, 24.4, 20.7, 20.6, 17.2 (2C), 16.2, 15.9, 15.0, 14.3, 12.8; IR (film, acetone): 3392, 2961, 2928, 2858, 1726, 1708, 1611, 1562, 1456, 1377, 1270, 1211, 1158, 1129, 1092, 1013, 981, 940, 902, 831, 807, 658; HR-ESI-MS calc. 749.4963 for C_44_H_70_NaO_8_^+^ [M+Na]^+^, found 749.4961.


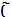

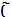

**Mycolactone F.** *R_f_* (EtOAc) = 0.54; ^1^H-NMR (400 MHz, acetone-d6): *δ* 7.36 (dd, *J* = 15.6, 0.5 Hz, 1H), 6.43 (s, 1H), 6.05 (s, 1H), 5.88 (d, *J* = 15.6 Hz, 1H), 5.59 (t, *J* = 7.3 Hz, 1H), 5.12 (d, *J* = 10.9 Hz, 1H), 5.04 (d, *J* = 9.3 Hz, 1H), 4.93 – 4.85 (m, 1H), 4.74 – 4.67 (m, 1H), 4.22 – 4.15 (m, 3H, OH), 4.10 – 4.04 (m, 1H, OH), 4.03 – 3.84 (m, 3H), 3.55 – 3.47 (m, 1H), 2.61 – 2.28 (m, 5H), 2.16 – 1.93 (m, 7H), 2.03 (d, *J* = 1.0 Hz, 3H), 2.01 (d, *J* = 1.0 Hz, 3H), 1.87 – 1.79 (m, 1H), 1.81 (s, 3H), 1.75 – 1.46 (m, 7H), 1.71 (s, 3H), 1.64 (d, *J* = 1.2 Hz, 3H), 1.44 – 1.34 (m, 1H), 1.15 (d, *J* = 2.0, 3H), 1.13 (d, *J* = 2.0 Hz, 3H), 0.98 (d, *J* = 6.7 Hz, 3H), 0.91 (d, *J* = 6.2 Hz, 3H), 0.89 (d, *J* = 6.7 Hz, 3H); ^13^C-NMR (100 MHz, acetone-d6): *δ* 173.3, 166.9, 151.1, 145.3, 139.0, 137.3, 134.6, 133.5, 132.9, 132.7, 131.3, 129.8, 123.9, 117.5, 79.3, 77.0, 76.3, 72.2, 69.0, 68.3, 46.5, 45.9, 44.4, 43.9, 43.8, 40.5, 37.9, 36.0, 35.4, 32.9, 31.4, 29.6 (obscured by solvent signal), 24.6, 24.5, 20.8, 20.5, 18.9, 17.3, 17.1, 16.2, 15.9, 15.0, 14.1; IR (film, acetone): 3412, 2963, 2931, 2360, 2343, 2330, 1718, 1685, 1457, 1375, 1254, 1162; HR-ESI-MS calc. 723.4806 for C_42_H_68_NaO_8_^+^ [M+Na]^+^, found 723.4811.


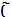

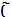

**(6*S*,7*S*,12*R*,*E*)-12-((2*S*,6*R*,7*R*,9*R*,*E*)-7,9-dihydroxy-4,6-dimethyldec-4-en-2-yl)-7,9-dimethyl-2-oxooxacyclododec-9-en-6-yl acetate (PG-119).** *R_f_* (*n*-hexane/EtOAc 1:1) = 0.36; [*α*]*_D_*^25^ = –25.3° (c = 1.07, CHCl_3_); ^1^H-NMR (400 MHz, acetone-d6): *δ* 5.10 (br s, *J* = 10.7 Hz, 1H), 5.04 (dd, *J* = 9.8, 1.0 Hz, 1H), 4.88 (ddd, *J* = 11.7, 5.0, 2.8 Hz, 1H), 4.62 – 4.56 (m, 1H), 4.20 (d, *J* = 3.3 Hz, 1H, OH), 4.18 – 4.15 (m, 1H, OH), 4.00 – 3.92 (m, 1H), 3.54 – 3.47 (m, 1H), 2.49 (dt, *J* = 14.0, 11.8 Hz, 1H), 2.43 – 2.35 (m, 2H), 2.16 – 1.86 (m, 7H), 1.99 (s, 3H), 1.82 (dd, *J* = 13.0, 8.8 Hz, 1H), 1.71 – 1.50 (m, 5H), 1.70 (s, 3H), 1.64 (d, *J* = 1.3 Hz, 3H), 1.43 – 1.33 (m, 1H), 1.13 (d, *J* = 6.1 Hz, 3H), 0.98 (d, *J* = 6.7 Hz, 3H), 0.88 (d, *J* = 6.7 Hz, 6H); ^13^C-NMR (100 MHz, acetone-d6): *δ* 173.3, 170.5, 137.4, 133.5, 131.3, 123.9, 79.5, 77.0, 76.3, 69.0, 46.5, 44.4, 43.9, 40.5, 36.0, 35.4, 32.6, 31.5, 29.6 (obscured by solvent signal), 24.6, 21.0, 20.7, 20.5, 17.1, 16.2, 15.9, 15.0; IR (film, acetone): 3425, 2962, 2930, 2873, 1727, 1455, 1436, 1370, 1330, 1240, 1165, 1130, 1092, 1019, 978, 957, 925, 885, 863, 834; HR-ESI-MS calc. 467.3367 for C_27_H_47_O_6_^+^ [M+H]^+^, found 467.3363.


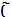

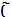

**(2*E*,4*E*)-((6*S*,7*S*,12*R*,*E*)-12-((2*S*,6*R*,7*R*,9*R*,*E*)-7,9-dihydroxy-4,6-dimethyldec-4-en-2-yl)-7,9-dimethyl-2-oxooxacyclododec-9-en-6-yl) hexa-2,4-dienoate (PG-120).** *R_f_* (*n*-hexane/EtOAc 1:1) = 0.47; [*α*]*_D_*^25^ = –40.3° (c = 1.10, CHCl_3_); ^1^H-NMR (400 MHz, acetone-d6): *δ* 7.24 (dd, *J* = 15.3 Hz, 10.0, 1H), 6.33 – 6.17 (m, 2H), 5.82 (d, *J* = 15.3 Hz, 1H), 5.12 (d, *J* = 10.7 Hz, 1H), 5.04 (d, *J* = 9.8 Hz, 1H), 4.93 – 4.86 (m, 1H), 4.71 – 4.65 (m, 1H), 4.22 – 4.16 (m, 2H, 2x OH), 4.00 – 3.92 (m, 1H), 3.54 – 3.47 (m, 1H), 2.55 – 2.44 (m, 1H), 2.44 – 2.33 (m, 2H), 2.16 – 1.91 (m, 7H), 1.90 – 1.79 (m, 1H), 1.84 (d, *J* = 5.8 Hz, 3H), 1.74 – 1.62 (m, 3H), 1.71 (s, 3H), 1.64 (d, *J* = 1.3 Hz, 3H), 1.62 – 1.52 (m, 2H), 1.44 – 1.34 (m, 1H), 1.14 (d, *J* = 6.2 Hz, 3H), 0.98 (d, *J* = 6.6 Hz, 3H), 0.90 – 0.87 (m, 6H); ^13^C-NMR (125 MHz, acetone-*d6*): *δ* 173.3, 166.7, 145.5, 139.9, 137.3, 133.4, 131.2, 130.7, 123.9, 120.3, 79.3, 76.9, 76.3, 68.9, 46.4, 44.4, 43.8, 40.5, 35.9, 35.4, 32.8, 31.4, 29.8 (obscured by solvent signal), 24.6, 20.8, 20.4, 18.6, 17.1, 16.2, 15.9, 15.0; IR (film, acetone): 2952, 2929, 2889, 2857, 1727, 1613, 1514, 1472, 1464, 1249, 1102, 1037, 1006, 836, 777; HR-ESI-MS calc. 519.3680 for C_31_H_51_O_6_^+^ [M+H]^+^, found 519.3671.


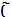

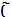

**(2*E*,8*E*,10*E*)-(6*S*,7*S*,12*R*,*E*)-12-((2*S*,6*R*,7*R*,9*R*,*E*)-7,9-dihydroxy-4,6-dimethyldec-4-en-2-yl)-7,9-dimethyl-2-oxooxacyclododec-9-en-6-yl 4,6,10-trimethylhexadeca-2,4,6,8,10-pentaenoate (PG-155).** *R_f_* (*n*-hexane/EtOAc 2:1) = 0.23; ***Z*-Δ^4’,5’^** **isomer:** ^1^H-NMR (400 MHz, acetone-d6): *δ* 7.92 (d, *J* = 15.8 Hz, 1H), 6.59 – 6.48 (m, 1H), 6.39 (s, *J* = 16.0 Hz, 1H), 6.32 (s, 1H), 6.14 (d, *J* = 10.9 Hz, 1H), 5.91 (d, *J* = 15.8 Hz, 1H), 5.67 – 5.59 (m, 1H), 5.12 (d, *J* = 10.5 Hz, 1H), 5.04 (d, *J* = 9.8, 1H), 4.99 – 4.75 (m, 1H), 4.75 – 4.57 (m, 1H), 4.23 – 4.12 (m, 2H, OH), 4.02 – 3.91 (m, 1H), 3.55 – 3.46 (m, 1H), 2.58 – 2.42 (m, 3H), 2.42 – 1.74 (m, 8H), 2.00 (s, 3H), 1.97 (s, 3H), 1.84 (s, 3H), 1.74 – 1.50 (m, 5H), 1.72 (s, 3H), 1.65 (d, *J* = 1.1 Hz, 3H), 1.50 – 1.23 (m, 9H), 1.14 (d, *J* = 6.1 Hz, 3H), 0.98 (d, *J* = 6.7 Hz, 3H), 0.95 – 0.80 (m, 9H); ^13^C-NMR (100 MHz, acetone-d6): *δ* 173.3, 166.9, 143.2, 142.0, 140.7, 137.3, 136.0, 135.7, 135.3, 133.7, 133.5, 131.8, 131.3, 123.8, 123.3, 119.5, 79.3, 77.0, 76.3, 69.0, 46.5, 44.4, 43.9, 40.5, 36.1, 35.4, 32.9, 32.3, 31.5, 30.0 (obscured by solvent signal), 29.7 (obscured by solvent signal), 29.1, 24.6, 23.2, 21.0, 20.7, 20.5, 17.6, 17.1, 16.2, 15.9, 15.0, 14.3, 12.5; ***E*-Δ^4’,5’^ isomer:** ^1^H-NMR (400 MHz, acetone-d6): *δ* 7.37 (d, *J* = 15.5 Hz, 1H), 6.59 – 6.48 (m, 1H), 6.46 (s, 1H), 6.43 (d, *J* = 15.6 Hz, 1H), 6.35 (d, *J* = 11.1 Hz, 1H), 5.88 (d, *J* = 15.5 Hz, 1H), 5.67 – 5.59 (m, 1H), 5.12 (d, *J* = 10.5 Hz, 1H), 5.04 (d, *J* = 9.8, 1H), 4.99 – 4.75 (m, 1H), 4.75 – 4.57 (m, 1H), 4.23 – 4.12 (m, 2H, OH), 4.02 – 3.91 (m, 1H), 3.55 – 3.46 (m, 1H), 2.58 – 2.42 (m, 3H), 2.42 – 1.74 (m, 11H), 2.07 (s, 3H), 1.83 (s, 3H), 1.74 – 1.50 (m, 5H), 1.72 (s, 3H), 1.65 (d, *J* = 1.1 Hz, 3H), 1.50 – 1.23 (m, 9H), 1.14 (d, *J* = 6.1 Hz, 3H), 0.98 (d, *J* = 6.7 Hz, 3H), 0.95 – 0.80 (m, 9H); ^13^C-NMR (100 MHz, acetone-d6): *δ* 173.3, 166.9, 151.3, 144.6, 141.1, 137.3, 136.7, 136.0, 135.7, 135.2, 133.5, 132.8, 131.3, 123.9, 123.3, 117.2, 79.3, 77.0, 76.3, 69.0, 46.5, 44.4, 43.9, 40.5, 36.0, 35.4, 32.9, 32.3, 31.4, 30.0 (obscured by solvent signal), 29.7 (obscured by solvent signal), 29.1, 24.6, 23.2, 20.8, 20.5, 17.1, 17.1, 16.2, 15.9, 15.0, 14.3, 14.3, 12.5; IR (film, acetone): 2957, 2928, 2856, 1728, 1708, 1612, 1559, 1456, 1378, 1330, 1303, 1269, 1249, 1211, 1158, 1131, 1014, 981; HR-ESI-MS calc. 717.5065 for C_44_H_70_NaO_6_^+^ [M+Na]^+^, found 717.5052.


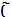

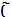

**(2*E*,8*E*,10*E*,12*S*,13*S*,15*S*)-(6*S*,7*S*,12*R*,*E*)-7,9-dimethyl-2-oxo-12-((2*S*,6*R*,7*R*,9*S*,*E*)-7,9,10-trihydroxy-4,6-dimethyldec-4-en-2-yl)oxacyclododec-9-en-6-yl 12,13,15-trihydroxy-4,6,10-trimethylhexadeca-2,4,6,8,10-pentaenoate (PG-165).** *R_f_* (EtOAc/MeOH 9:1) = 0.31; ***Z*-Δ^4’,5’^** **isomer:** ^1^H-NMR (400 MHz, acetone-d6): *δ* 7.92 (d, *J* = 15.3 Hz, 1H), 6.76 – 6.57 (m, 1H), 6.53 – 6.26 (m, 2H), 6.16 (d, *J* = 10.7 Hz, 1H), 5.94 (d, *J* = 15.3 Hz, 1H), 5.65 – 5.54 (m, 1H), 5.19 – 5.09 (m, 1H), 5.06 (d, *J* = 10.0 Hz, 1H), 4.99 – 4.83 (m, 1H), 4.80 – 4.61 (m, 1H), 4.36 – 4.23 (m, 1H), 4.23 – 4.14 (m, 3H, 3 x OH), 4.05 – 3.94 (m, 2H, 1 x OH), 3.92 – 3.76 (m, 2H, 1 x OH), 3.75 – 3.37 (m, 5H, 1 x OH), 2.59 – 2.30 (m, 3H), 2.18 – 1.46 (m, 21H), 2.09 (s, 3H), 1.71 (d, *J* = 4.4 Hz, 3H), 1.64 (d, *J* = 1.0 Hz, 3H), 1.45 – 1.34 (m, 1H), 1.14 – 1.10 (m, 3H), 0.99 (d, *J* = 6.7 Hz, 3H), 0.93 – 0.84 (m, 6H); ^13^C-NMR (125 MHz, acetone-d6): *δ* 173.4, 167.0, 143.2, 141.8, 139.9, 137.3, 137.3, 134.9, 134.8, 134.6, 133.4, 132.1, 131.1, 125.1, 123.8, 119.5, 79.3, 76.3 (2C), 75.7, 73.5, 72.3, 67.7, 67.3, 46.3, 44.3, 41.8, 40.4, 38.3, 36.0, 35.4, 32.8, 31.3, 29.7 (obscured by solvent signal), 24.2, 21.0, 20.8, 20.5, 17.6, 17.2, 16.2, 15.9, 15.0, 13.4; ***E*-Δ^4’,5’^** **isomer:** ^1^H-NMR (400 MHz, acetone-d6): *δ* 7.37 (d, *J* = 15.5 Hz, 1H), 6.76 – 6.57 (m, 1H), 6.53 – 6.26 (m, 3H), 5.89 (d, *J* = 15.5 Hz, 1H), 5.65 – 5.54 (m, 1H), 5.19 – 5.09 (m, 1H), 5.06 (d, *J* = 10.0 Hz, 1H), 4.99 – 4.83 (m, 1H), 4.80 – 4.61 (m, 1H), 4.36 – 4.23 (m, 1H), 4.23 – 4.14 (m, 3H, 3 x OH), 4.05 – 3.94 (m, 2H, 1 x OH), 3.92 – 3.76 (m, 2H, 1 x OH), 3.75 – 3.37 (m, 5H, 1 x OH), 2.59 – 2.30 (m, 3H), 2.18 – 1.46 (m, 18H), 2.02 (s, 3H), 1.98 (s, 3H), 1.71 (d, *J* = 4.4 Hz, 3H), 1.64 (d, *J* = 1.0 Hz, 3H), 1.45 – 1.34 (m, 1H), 1.14 – 1.10 (m, 3H), 0.99 (d, *J* = 6.7 Hz, 3H), 0.93 – 0.84 (m, 6H); ^13^C-NMR (125 MHz, acetone-d6): *δ* 173.4, 167.0, 151.2, 144.4, 140.3, 137.3, 136.2, 135.3, 134.8, 133.4, 133.1, 131.1, 125.1, 123.8, 117.3, 79.3, 76.3 (2C), 75.7, 73.5, 72.3, 67.7, 67.3, 46.3, 44.3, 41.8, 40.4, 38.3, 36.0, 35.4, 32.8, 31.3, 29.7 (obscured by solvent signal), 24.2, 20.8, 20.5, 17.2, 17.1, 16.2, 15.9, 15.0, 14.3, 13.4; IR (film, acetone): 3403, 2929, 2360, 2340, 1716, 1456, 1252, 1160, 668; HR-ESI-MS calc. 781.4861 for C_44_H_70_NaO_10_^+^ [M+Na]^+^, found 781.4856.


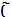

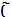

**(2*E*,8*E*,10*E*,12*S*,13*S*,15*S*)-(6*S*,7*S*,12*R*,*E*)-12-((2*S*,6*R*,7*R*,9*S*,*E*)-10-acetoxy-7,9-dihydroxy-4,6-dimethyldec-4-en-2-yl)-7,9-dimethyl-2-oxooxacyclododec-9-en-6-yl 12,13,15-trihydroxy-4,6,10-trimethylhexadeca-2,4,6,8,10-pentaenoate (PG-157).** *R_f_* (EtOAc/MeOH 9:1) = 0.52; ***Z*-Δ^4’,5’^** **isomer:** ^1^H-NMR (400 MHz, acetone-d6): *δ* 7.92 (d, *J* = 15.4 Hz, 1H), 6.69 – 6.60 (m, 1H), 6.53 – 6.27 (m, 2H), 6.16 (d, *J* = 10.6 Hz, 1H), 5.94 (d, *J* = 15.4 Hz, 1H), 5.64 – 5.54 (m, 1H), 5.13 (d, *J* = 9.1 Hz, 1H), 5.06 (d, *J* = 10.1 Hz, 1H), 4.96 – 4.83 (m, 1H), 4.77 – 4.65 (m, 1H), 4.50 (s, 1H, OH), 4.35 – 3.82 (m, 9H, 4 x OH), 3.74 –3.47 (m, 2H), 2.58 – 2.33 (m, 3H), 2.18 – 1.31 (m, 31H), 1.65 (d, *J* = 1.1 Hz, 3H), 1.21 – 1.09 (m, 3H), 0.99 (d, *J* = 6.6 Hz, 3H), 0.95 – 0.80 (m, 6H); ^13^C-NMR (100 MHz, acetone-d6): δ 173.3, 171.1, 166.9, 143.1, 141.8, 140.0, 137.4, 137.3, 134.9, 134.8, 134.6, 133.7, 132.1 (assigned by 125 MHz experiments), 131.0, 125.2, 123.8, 119.6 (assigned by 125 MHz experiments), 79.3, 76.4, 76.2, 75.7, 72.3, 70.5, 69.2, 67.7, 46.3, 44.3, 41.8, 40.4, 38.3, 36.0, 35.5, 32.8, 31.3, 29.5 (obscured by solvents signal), 24.2, 21.1, 21.0, 20.8, 20.6, 17.6, 17.1, 16.3, 15.9, 15.0, 13.4; ***E*-Δ^4’,5’^** **isomer:** ^1^H-NMR (400 MHz, acetone-d6): *δ* 7.37 (d, *J* = 15.6 Hz, 1H), 6.69 – 6.60 (m, 1H), 6.53 – 6.27 (m, 3H), 5.90 (d, *J* = 15.6 Hz, 1H), 5.64 – 5.54 (m, 1H), 5.13 (d, *J* = 9.1 Hz, 1H), 5.06 (d, *J* = 10.1 Hz, 1H), 4.96 – 4.83 (m, 1H), 4.77 – 4.65 (m, 1H), 4.50 (s, 1H, OH), 4.35 – 3.82 (m, 9H, 4 x OH), 3.74 –3.47 (m, 2H), 2.58 – 2.33 (m, 3H), 2.18 – 1.31 (m, 28H), 2.09 (s, 3H), 1.65 (d, *J* = 1.1 Hz, 3H), 1.21 – 1.09 (m, 3H), 0.99 (d, *J* = 6.6 Hz, 3H), 0.95 – 0.80 (m, 6H); ^13^C-NMR (100 MHz, acetone-d6): *δ* 173.3, 171.1, 166.9, 151.2, 144.3, 140.4, 137.4, 137.3, 136.2, 135.3, 134.8, 133.7, 133.2, 131.0, 125.2, 123.8, 117.4, 79.3, 76.4, 76.2, 75.7, 72.3, 70.5, 69.2, 67.7, 46.3, 44.3, 41.8, 40.4, 38.3, 36.0, 35.5, 32.8, 31.3, 29.5 (obscured by solvents signal), 24.2, 21.1, 20.8, 20.6, 17.1, 17.1, 16.3, 15.9, 15.0, 14.3, 13.4; IR (film, acetone): 3394, 2962, 2930, 2874, 1706, 1610, 1562, 1454, 1438, 1376, 1330, 1302, 1248, 1158, 1131, 1089, 1037, 1012, 981, 936, 845; HR-ESI-MS calc. 823.4967 for C_46_H_72_NaO_11_^+^ [M+Na]^+^, found 823.4963.


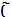

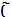

**(2*E*,8*E*,10*E*,12*S*,13*S*,15*S*)-(6*S*,7*S*,12*R*,*E*)-12-((2*S*,6*R*,7*R*,9*S*,*E*)-10-((butylcarbamoyl)oxy)-7,9-dihydroxy-4,6-dimethyldec-4-en-2-yl)-7,9-dimethyl-2-oxooxacyclododec-9-en-6-yl 12,13,15-trihydroxy-4,6,10-trimethylhexadeca-2,4,6,8,10-pentaenoate (PG-182).** *R_f_* (EtOAc) = 0.17; ***Z*-Δ^4’,5’^** **isomer:** ^1^H-NMR (400 MHz, acetone-d6): *δ* 7.92 (d, *J* = 15.3 Hz, 1H), 6.77 – 6.53 (m, 1H), 6.52 – 6.30 (m, 2H), 6.28 – 6.09 (m, 2H, 1 x NH), 5.94 (d, *J* = 15.9 Hz, 1H), 5.69 – 5.50 (m, 1H), 5.13 (d, *J* = 10.1 Hz, 1H), 5.04 (d, *J* = 9.6 Hz, 1H), 4.96 – 4.82 (m, 1H), 4.79 – 4.67 (m, 1H), 4.57 (s, 1H, OH), 4.35 – 3.81 (m, 9H, 4 x OH), 3.67 (ddd, *J* = 9.6, 6.2, 3.3 Hz, 1H), 3.62 – 3.48 (m, 1H), 3.21 – 3.04 (m, 2H), 2.60 – 2.32 (m, 3H), 2.20 – 1.20 (m, 35H), 1.18 – 1.08 (m, 3H), 1.02 – 0.95 (m, 3H), 0.95 – 0.77 (m, 9H); ^13^C-NMR (100 MHz, acetone-d6): *δ* 173.4, 167.0, 157.7 (assigned in 125 MHz experiment), 143.2, 141.9, 140.0, 137.4, 137.3, 134.9, 134.9, 134.7, 133.7, 132.1, 131.1, 125.2, 123.9, 119.6, 79.4, 76.3, 76.1, 75.8, 72.4, 71.5, 69.6, 67.7, 46.3, 44.4, 41.9, 41.3, 40.4, 38.5, 36.0, 35.4, 32.9, 32.8, 31.4, 29.3 (obscured by solvent signal), 24.2, 21.0, 20.8, 20.6, 20.6, 17.6, 17.1, 16.3, 15.9, 15.0, 14.1, 13.4; ***E*-Δ^4’,5’^** **isomer:**^1^H-NMR (400 MHz, acetone-d6): *δ* 7.37 (d, *J* = 15.6 Hz, 1H), 6.77 – 6.53 (m, 1H), 6.52 – 6.30 (m, 3H), 6.28 – 6.09 (m, 1H, NH), 5.90 (d, *J* = 15.6 Hz, 1H), 5.69 – 5.50 (m, 1H), 5.13 (d, *J* = 10.1 Hz, 1H), 5.04 (d, *J* = 9.6 Hz, 1H), 4.96 – 4.82 (m, 1H), 4.79 – 4.67 (m, 1H), 4.57 (s, 1H, OH), 4.35 – 3.81 (m, 9H, 4 x OH), 3.67 (ddd, *J* = 9.6, 6.2, 3.3 Hz, 1H), 3.62 – 3.48 (m, 1H), 3.21 – 3.04 (m, 2H), 2.60 – 2.32 (m, 3H), 2.20 – 1.20 (m, 35H), 1.18 – 1.08 (m, 3H), 1.02 – 0.95 (m, 3H), 0.95 – 0.77 (m, 9H); ^13^C-NMR (100 MHz, acetone-d6): *δ* 173.4, 167.0, 157.7 (assigned in 125 MHz experiment), 151.3, 144.4, 140.4, 137.4, 137.3, 136.2, 135.3, 134.9, 133.7, 133.2, 131.1, 125.2, 123.9, 117.4, 79.4, 76.3, 76.1, 75.8, 72.4, 71.5, 69.6, 67.7, 46.3, 44.4, 41.9, 41.3, 40.4, 38.5, 36.0, 35.4, 32.9, 32.8, 31.4, 29.3 (obscured by solvent signal), 24.2, 20.8, 20.6, 20.6, 17.1, 17.1, 16.3, 15.9, 15.0, 14.3, 14.1, 13.4; IR (film, acetone): 3368, 2960, 2929, 2871, 1705, 1611, 1559, 1457, 1383, 1330, 1303, 1251, 1159, 1132, 1093, 1014, 983, 939, 846; HR-ESI-MS calc. 880.5545 for C_49_H_79_NNaO_11_^+^ [M+Na]^+^, found 880.5537.


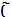

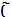


# ^1^H- and ^13^C-NMR Spectra of Mycolactone Derivatives

**PG-119**
